# Arp2/3: Structural Insights into a Primary Engine of Cell Motility

**DOI:** 10.1371/journal.pbio.0030411

**Published:** 2005-11-08

The capacity for directed motion arises from core cellular processes shared by organisms from protozoa to primates, indicating an ancient origin over a billion years ago. Amoeba foraging for food, pathogenic microbes invading host tissue, host immune cells ingesting those pathogens—all depend on the same core motility components. Actin, the most abundant protein in eukaryotic cells (cells with nuclei), lies at the heart of the motility machinery. Individual actin proteins assemble into polymers and organize into long filaments that form intricate networks under the direction of a seven-subunit protein complex called the actin-related protein (Arp) 2/3 complex. As actin polymerization occurs, newly formed daughter filaments grow out at an angle from mother filaments, much like branches sprout from a tree trunk. The place where daughter filaments bud from a mother filament (branching nucleation) is called the branch junction.

Branch formation requires Arp2/3 activation, which occurs when the Arp2/3 complex binds to nucleation promoting factors (which includes a family of proteins called WASp), molecules of adenosine triphosphate (ATP)—a molecule that fuels most energy-requiring processes—and already-existing mother filaments. The details of branch junction formation have remained obscure, though nucleation models have proposed that Arp2 and Arp3 assemble around the mother filament and form a template for actin subunits to nucleate from. While the atomic structure of the inactive Arp2/3 complex (previously determined) shows that the two actin-related proteins resemble actin enough to form a template for nucleation, the spatial relationship between the proteins in the inactive complex does not match that in actin filaments.

Current models postulate that Arp2/3 complex activation triggers a change in spatial relationship so that Arp2 and Arp3 resemble actin monomers in a filament; but without evidence on the structure of the Arp2/3 subunits at the branch junctions, these models have to assume that this is how nucleation occurs. In a new study, Coumaran Egile et al. combine electron microscopy, genetic labeling, and computational analysis to resolve the structure of the Arp2/3 complex at the branch junction to a resolution of one nanometer (that's one billionth of a meter), and demonstrate that the Arp2/3 template assumption is correct.

To study the mechanics of branched actin nucleation by the Arp2/3 complex, Egile et al. assembled actin branches in test tubes and observed the action at the molecular level. To do this, the researchers tagged the different Arp2/3 subunits with labels that can be detected with electron microscopy, allowing them to determine the location of the proteins. Using this approach, they introduced different nucleation promotion factors—WASp proteins as well as cortactin (an Arp2/3 activator that is found near the inner cell membrane)—and compared the resulting branch junction formations. Only cortactin was found at the branch junction, supporting the model that WASp activators transiently bind, activate, and release Arp2/3 after branch formation. Cortactin, on the other hand, may stay behind to help stabilize the interaction between Arp2/3 and the mother filament. [Fig pbio-0030411-g001]

**Figure pbio-0030411-g001:**
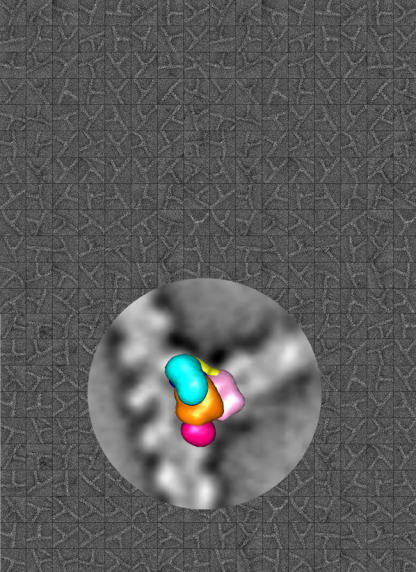
The actin-related protein (Arp) 2/3 complex provides a template for new actin filaments to branch off from a mother filament

After genetically engineering yeast to express fluorescently labeled versions of the different subunits, Egile et al. observed the complexes' nucleation activities and located their position in the branch junction. The likely orientation of the subunits at the branch junction was determined with computational modeling. Given the position of the subunits and the number of possible combinations at this site, the authors used the crystal structure of the inactive Arp2/3 complex to map all the possible orientations. Only one cluster of orientations satisfied the constraints: Arp2 and Arp3, associated with the fast-growing end in an actin filament, facing away from the mother filament and toward the daughter filament.

Though Arp2 and Arp3 orientations would have to change upon activation to support daughter filament growth, the authors argue that the change would not disrupt the overall architecture of the complex. Rotating the subunits to reflect their activated conformations places Arp3 next to the mother filament and Arp2 farthest away. In this orientation, the longest axis of the complex aligns perpendicular to the mother filament (in all previous models, they align parallel), an arrangement that could provide stability at the branch junction, by taking advantage of protein interactions on either side of the mother filament.

Altogether, these results provide conclusive evidence for the starting assumption of a nucleation model in which Arp2 and Arp3 form a template at the branch junction that triggers daughter filament growth. And with the help of the subunit map presented here, researchers can further dissect the molecular mechanisms of actin branch nucleation and elucidate the dynamics of cellular motion. *—Liza Gross*

